# Multi-Scale Low-Entropy Method for Optimizing the Processing Parameters during Automated Fiber Placement

**DOI:** 10.3390/ma10091024

**Published:** 2017-09-03

**Authors:** Zhenyu Han, Shouzheng Sun, Hongya Fu, Yunzhong Fu

**Affiliations:** School of Mechatronics Engineering, Harbin Institute of Technology, No.92, Xidazhi Street, Harbin 150001, China; hanzy@hit.edu.cn (Z.H.); sunshouzheng.hit@outlook.com (S.S.); hongyafu@hit.edu.cn (H.F.)

**Keywords:** carbon fiber-reinforced composites, automated fiber placement, multi-scale analysis, low-entropy method, processing optimization

## Abstract

Automated fiber placement (AFP) process includes a variety of energy forms and multi-scale effects. This contribution proposes a novel multi-scale low-entropy method aiming at optimizing processing parameters in an AFP process, where multi-scale effect, energy consumption, energy utilization efficiency and mechanical properties of micro-system could be taken into account synthetically. Taking a carbon fiber/epoxy prepreg as an example, mechanical properties of macro–meso–scale are obtained by Finite Element Method (FEM). A multi-scale energy transfer model is then established to input the macroscopic results into the microscopic system as its boundary condition, which can communicate with different scales. Furthermore, microscopic characteristics, mainly micro-scale adsorption energy, diffusion coefficient entropy–enthalpy values, are calculated under different processing parameters based on molecular dynamics method. Low-entropy region is then obtained in terms of the interrelation among entropy–enthalpy values, microscopic mechanical properties (interface adsorbability and matrix fluidity) and processing parameters to guarantee better fluidity, stronger adsorption, lower energy consumption and higher energy quality collaboratively. Finally, nine groups of experiments are carried out to verify the validity of the simulation results. The results show that the low-entropy optimization method can reduce void content effectively, and further improve the mechanical properties of laminates.

## 1. Introduction

**Figure 1:**
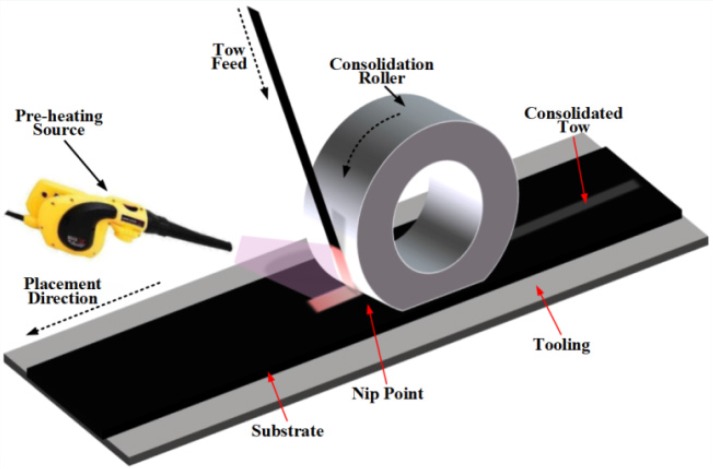
Automated Fiber Placement (AFP) process.

A popular type of prepregs are laminated and then cured to be fiber-reinforced composites [1,2,3,4], which have a number of advantages in comparison to metal-based structures, including the fact that they offer lightweight and stiff surfaces, which resist corrosion well and are also believed to have a relatively long fatigue life [5]. Hence, composite materials are now increasingly used as large and complex primary structures in aeronautics industry. However, their well-known manufacturing weaknesses, such as pores and micro-crack, drive many companies to develop new methods to obtain better part quality. The recent introduction of airplanes with parts made by the Automated Fiber Placement (AFP) process has increased the demand for this method. An AFP machine consists of a computer controlled robotic arm with a placement head end effector that lays bands of prepreg strips onto a mold to construct the lay-up. The bands are made with 8–32 prepreg strips, called tows, which are aligned side-by-side by the placement head. For each ply, the machine accurately places the bands on the mold respecting the proper ply angles and covering technique. The part is then placed in an autoclave to polymerize the resin material and consolidate the plies [6]. The Automated Fiber Placement process is shown in Figure 1.

Material properties have a multi-scale effect, which is no exception for composites fabricated by AFP. On the one hand, the formation and expansion process of manufacturing defects for prepreg have the multi-scale effect in AFP, which is a “long” creep process and includes different spatiotemporal scales [7]. This statement also works from the physical point of view that cracks form at the atomic scale, extend to the macroscopic level, are irreversible, and travel far from equilibrium [8]. On the other hand, mechanical properties also have the multi-scale effect during AFP process. Small-scale mechanical behaviors are the latent phenomenon of large-scale performance. However, small-scale mechanical properties cannot be completely reflected in large-scale performance that could be measured traditionally. Unfortunately, these multi-scale problems remain a compelling challenge until now [9,10,11,12,13,14]. Although much research has been done on the mechanical properties of composites during service life [15,16,17], little work focuses on the one under different scales during AFP process.

Evaluation of energy is an important means to study the mechanical properties of multi-scale. Entropy is adopted to assess energy quality in microscopic particles under a certain state of system [18]. Low-entropy determines that the system has higher energy utilization efficiency, which is known as high-quality energy [19]. Many studies on entropy have been performed in mathematics, astronomy, materials science, etc. [20,21,22]. Chakraborty employed adsorption uptake data of activated carbons and some non-polar gases to calculate the enthalpy and entropy of adsorption in pressure–temperature–uptake coordinate systems [23]. Jafarmadar investigated the flow, heat transfer and entropy generation based on the second law of thermodynamics [24]. Ahmed utilized a numerical study to clarify heat transfer characteristics, effectiveness and entropy generation for a bundle of wingshaped-tubes attached to longitudinal fins at downstream side [25]. However, little research has been dedicated to the optimization of processing parameters by using low-entropy method.

The aim of this article is to optimize processing parameters in AFP through a multi-scale low-entropy design method, which could achieve better fluidity, stronger adsorption, lower energy consumption and higher energy quality collaboratively. The rest of this paper is organized as follows. In Section 2, a novel multi-scale low-entropy design method is proposed based on concurrent/sequential multi-scale analysis method, energy transfer model, Finite Element Method (FEM) and Molecular Dynamics (MD) method. In Section 3, mechanical properties of macro–meso-scale are calculated. A multi-scale energy transfer model is established through composite meso-mechanics and classical mechanics. Microscopic characteristics then are obtained under different processing parameters, including entropy–enthalpy values, interface adsorbability and matrix fluidity. Low-entropy region is found out in terms of the entropy–enthalpy values and different multi-scale mechanical properties in Section 4. Lastly, nine groups of experiments are implemented to verify the validity of the simulation results.

## 2. Multi-Scale Low-Entropy Method

**Figure 2:**
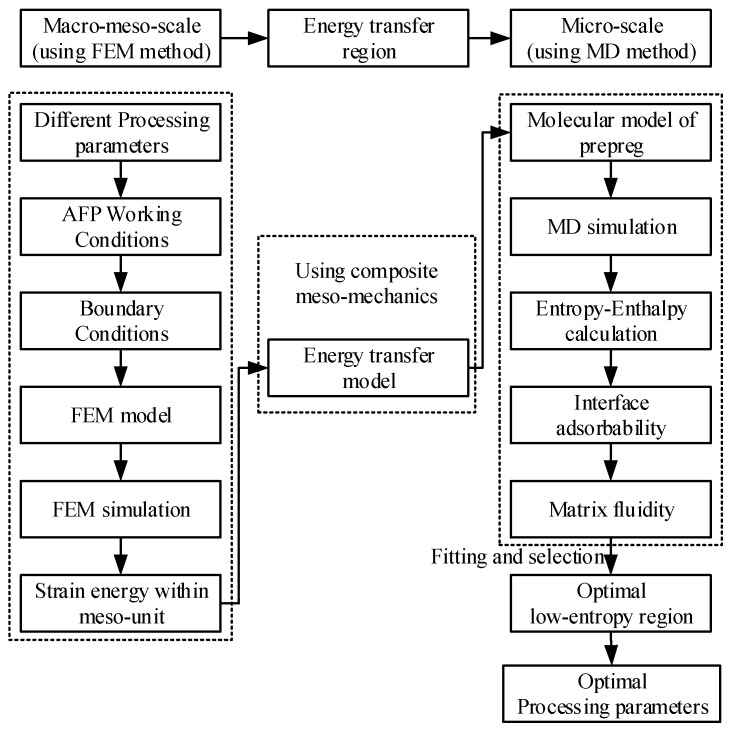
Multi-scale low-entropy design method.

Based on concurrent/sequential multi-scale analysis method [26], energy transfer model, finite element method and Molecular Dynamics (MD) method, a multi-scale low-entropy design method is proposed, as shown in Figure 2, which is also the general idea in this paper. This method is applicable for band-shape structure of prepreg.

In Figure 2, energy characteristics of macro–meso-scale are firstly obtained by using FEM simulation under different processing parameters and AFP working conditions. Via multi-scale energy transfer model, strain energy of meso-scale is converted into potential energy of micro-scale that could serve as boundary conditions of MD simulation. Furthermore, entropy–enthalpy values are calculated by statistical entropy–enthalpy algorithm and MD simulation under different processing parameters. Simultaneously, interface adsorbability and matrix fluidity of micro-scale are obtained by MD simulation. Relational data of Enthalpy–entropy with adsorbability and fluidity are fit to obtain the surfaces and contour maps, respectively. An optimal low-entropy region is then found, which considers low-entropy, low-energy (i.e., low-enthalpy) and better microscopic properties (i.e., better adsorbability and fluidity) collaboratively.

## 3. Multi-Scale Analysis

### 3.1. Macro-Scale and Meso-Scale

In this subsection, mechanical properties of macro-scale and meso-scale are obtained based on Finite Element Method. Firstly, Bisphenol A epoxy matrix prepreg is used to serve as an analytical object. Mechanical properties of a carbon fiber/epoxy prepreg are shown in Table 1.

**Figure 3:**
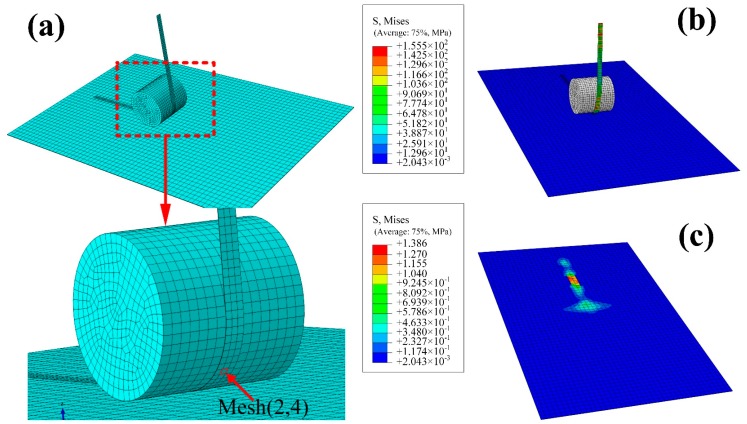
FEM model and stress distribution at the laying speed of 27m/min and compaction force of 200KPa: (**a**) FEM model and mesh(2,4); (**b**) stress distribution of the entire model; and (**c**) stress distribution of the laminates.

FEM model is established (Figure 3) in this study, which includes roller, prepreg tow and laminates in high-speed AFP. The laying speed is set as 27 m/min, 30 m/min, 33 m/min and 36 m/min, respectively. The compaction force is considered to be 150 KPa, 200 KPa, 250 KPa and 300 KPa, respectively. The levels of laying speed and compaction are cross-linked, thus 16 groups of boundary conditions could be obtained. In this study, the simulation time is set to be 125 μs. The bottom of laminates, which are formed by four unidirectional layers, is fully constrained. In addition, the actual length of lay-up tow is 280 mm, and contact type is set as rough between the laying tow and the laminates. To analyze the meso-mechanical characteristics of a unit in the laying tow, mesh(2,4) is chosen to serve as an analytical object that draws in Figure 3a. Partial results are shown in Figure 3b,c.

In Figure 3, the distance from the beginning of rolling to the ending of rolling through a unit is called roll nip of a unit here. The mesh(2,4) is compressed by roller directly, which is an important feature of meso-unit in roll nip region. Hence, it is reasonable that a micro-system within the mesh(2,4) is chosen to calculate microscopic properties.

### 3.2. Multi-Scale Energy Transfer Model

Due to multi-scale effect of mechanical properties and the distinctive structure of monolayer prepreg tow in an AFP process, multi-scale analysis method could be used to study the mechanical properties of laying tow. However, the difficulty is how to establish the relationship among the mechanical properties of different scales. To solve this problem, a kind of “intermediate”, which could link up with the different scales, must be found out. Energy and their transfer waves could be regarded as the “intermediate” because of energy conservation law among different scales in a time domain. Some publications showed that establishment of energy transfer model is the key to solving the multi-scale effect [27,28,29]. In general, there are two difficulties. One is the incoordination of constitutive relation between finite element region and molecular dynamics region, and the other is adverse effect of the incoordinationon energy wave transfer. MADD method (i.e., macroscopic, atomistic, and ab-initio dynamics) [30] could be known as the origin of the calculation method of multi-scale energy transfer region, which combined with tight-binding method, finite element method and molecular dynamics (MD). Mechanical properties within quantum, atomic and macroscopic scales are calculated simultaneously by the MADD method, but its drawback is that the design of energy transfer region cannot take the reflection of energy waves into consideration. If so, the reflected energy is reserved into FEM region or MD region, even making the regions melt away entirely [31]. Focusing on the problem, one-dimensional model [32], Cauchy–Born rule and quasi-continuous multi-scale analysis method (QC) [33] were developed. However, these works suffer from some limitations, e.g., these methods are only applicable for some certain special situations. Three-dimensional model of the impurities cannot be resolved. Thus, a novel multi-scale energy transfer model that is applicable for orthotropic materials should be built. The energy transfer model is further studied based on Reference [34].

**Figure 4:**
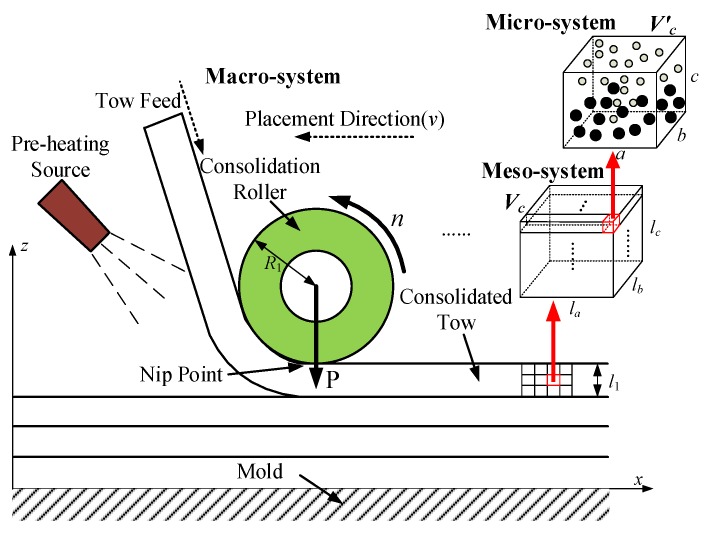
AFP process and multi-scale effects.

Considering macro–meso-scale, the prepreg tow is forced to develop into elastic and plastic deformation subjected to multiple laying loads, and strain energy is generated during this process. With the continuous laying process, the strain energy could be continued to accumulate and dissipate. It is worth noting that this process is approximately “parallel” developing with micro-scale, and then the phenomena of spall and creep are driven, which could form the earliest defects. If the propagation time of stress wave is negligible because of this “parallel” development, the strain energy transfer of different scales could be known as “static” process at some point. For example, provided that macro–meso-system contains the micro-system, the change of strain energy within the macro–meso-scale could affect molecular potential energy within the micro-scale in realtime. This interrelation of the multi-scale system is shown in Figure 4.

The composite mesoscopic mechanics methodology is used to calculate the strain energy of meso-unit. In addition, there are the following assumptions:

(1) Monolayer composite is macro uniform, orthotropic, and no initial stress material.
(2) Fibers are transversely isotropic, uniform, and regularly arranged.
(3) Matrix is uniform and isotropic.

There are *n* meso units within the macro-system that are assumed. Boundary conditions and loads are set up. Inelastic properties of orthotropic composite along the main material direction can be expressed in terms of strain energy density *u*_0_, which is obtained in Equation (1).

(1) $$e_{i}=\frac{\partial u_{0}}{\partial s_{i}}$$

Strain energy can be expanded to polynomial of *σ* under zero initial stress.

(2) $$u_{0}=\frac{1}{C_{ij}}\sigma_{i}\sigma_{j}+\frac{1}{3}\frac{1}{C_{ijk}}\sigma_{i}\sigma_{j}\sigma_{k}+\frac{1}{4}\frac{1}{C_{ijkl}}\sigma_{i}\sigma_{j}\sigma_{k}\sigma_{l}$$

In Equation (2), non-zero constant of *C_ij_* describes the part of the linear deformation. *C_ijk_* and *C_ijkl_* denote the part of the nonlinear deformation.

Relational model between internal energy increment and the strain energy density within the meso-unit is obtained in Equation (3), based on the classical mechanics.

(3) $$dU=\underset{V}{∭}du_{0}dV$$

There is a hypothesis that the internal energy of meso-unit is zero in the initial placement. Thus, the internal energy increment of system is approximately equal to the potential energy. The meso-system could not be suffered from the external work simultaneously. The superposition of the internal energy within *n* units is used to obtain the total strain energy *Q*(*U*) of macro-system:

(4) $$Q(U)=\sum_{m=1}^{n}U_{m}=\sum_{m=1}^{n}\underset{V_{m}}{∭}\delta u_{0}dV_{m}$$

A meso-unit could be regarded as homogeneous distribution of energy within the unit. The macro-system could not be set as energy homogeneous body because the energy difference within different regions in the macro-system is significant. The scaling of energy within different scales is obtained using the homogenization method, which is shown in Equation (5):

(5) $$\kappa_{1}=\frac{U_{c}}{Q(U)}=\frac{\underset{V_{c}}{∭}\delta u_{0}dV_{c}}{\sum_{m=1}^{n}\underset{V_{m}}{∭}\delta u_{0}dV_{m}};\kappa_{2}=\frac{{U^{\prime }}_{c}}{U_{c}}=\frac{{V^{\prime }}_{c}}{V_{c}}$$

In Equation (5), there are many *κ*_1_ in the macro-system, which could assess the homogeneity of local energy distribution within macro-scale. *κ*_2_ could be obtained by using the homogenization method that mesoscopic energy is uniformly divided by a number of micro-systems. From the above equation, the smaller the volume of meso-unit is or the larger the volume of micro-system is, the more accurately energy within the micro-system can be calculated. There is a special limiting case: the calculation of energy has no error when the volume of meso-unit is as large as that of micro-system. However, it causes the following problems. On the one hand, it could lead to the complexity of macro calculation. For example, in finite element analysis, calculation time is determined by the number and size of meshes. Smaller or denser meshes would cost a lot of time. Therefore, it is the lack of reason in math that atomic distance variables are used in the continuum region. On the other hand, it would be meaningless for finite element constitutive equation that meso-unit is reduced to microscopic size. Consequently, the partition of finite elements tries to be in the range of meso-scale, so that microscopic energy transfer errors could be reduced. The above formulas are applicable for orthotropic materials.

### 3.3. Boundary Condition of Micro-Scale

The calculation results of macro–meso-scale could be acted as boundary conditions for micro-system by using multi-scale energy model. Some boundary conditions such as *S*_0_ and*κ*_2_ are calculated in accordance with Equations (1)–(5), which are shown in Table 2.

**Figure 5:**
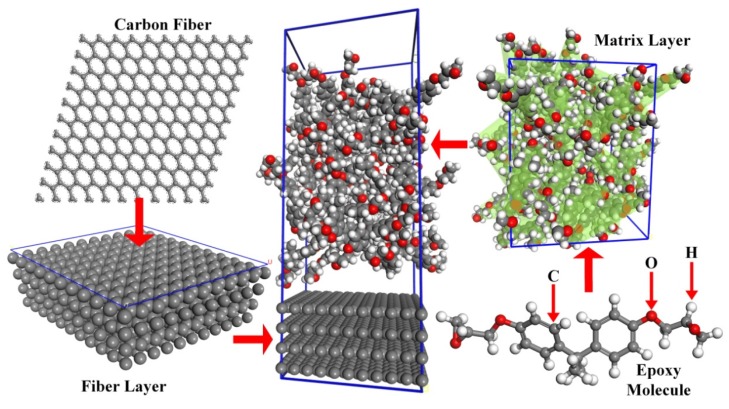
Particle swarm configuration of micro-system.

Molecular structure of a Bisphenol A epoxy resin is established using chemical formula and molecular weight (340). Amorphous structure of 40 epoxy resin molecules (the density is 1.2 g/cm^3^) are then established and optimized. Carbon fiber layer and its supercell is established (the density is 1.78 g/cm^3^).The micro-system (24.2 Å × 24.2 Å × 61.1 Å) of prepreg tow is further built, which combined epoxy resin layer with carbon fiber layer. The specific steps for stability optimization of micro-system are as follows. Firstly, the micro-system is optimized to guarantee as table system that contains the minimum total energy. Secondly, relaxation process of this stable system is implemented by using NVT ensemble with the temperature of 298 K, at the time step of 1 fs and the simulation time of 200 ps, which can obtain the balanced configuration of micro-system. Finally, density of new micro-system is calculated to prove the accuracy of the system. Density of micro-system is 1.51 g/cm^3^ and the fact is 1.49 g/cm^3^. The results show that the density error is 1.32%. The final micro-system is shown in Figure 5.

**Figure 6:**
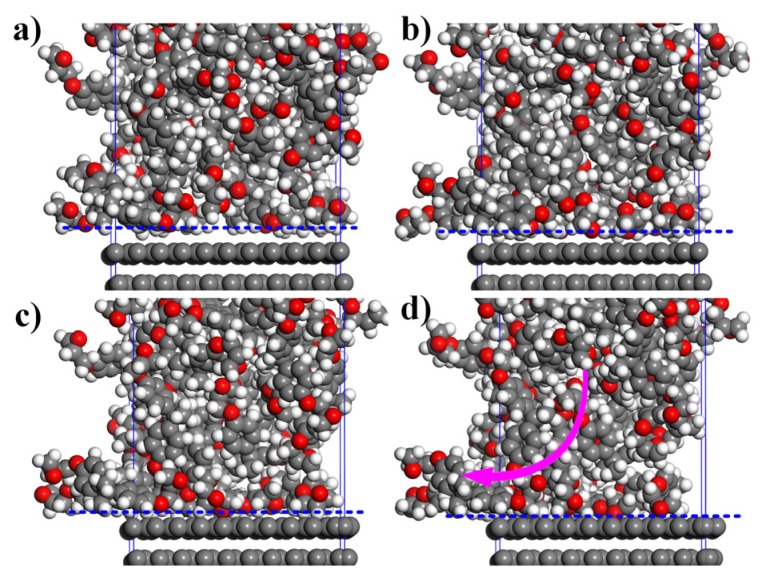
The adsorption process at the compaction force of 300KPa, the laying speed of 30 m/min and the pre-heating temperature of 333.15 K: (**a**) 0 ps; (**b**) 10 ps; (**c**) 20 ps; and (**d**) 30 ps (the arrow refers to the adsorption direction).

The micro-system is uniformly heated without taking heat conduction and thermal diffusion into consideration, and pre-heating factor is increased in the micro-system. Some experiences show that the highest pre-heating temperature about 350 K or so may be applied in epoxy resins. Thus, the pre-heating temperature is set as 313.15 K, 323.15 K, 333.15 K and 343.15 K respectively. Then the 64 groups of MD simulation parameters can be obtained by linking to the 16 groups of FEM conditions. Strain energy in roll nip aimed at mesh(2,4) are extracted to input the micro-system through energy transfer model (Equations (2), (3) and (5)). It explains specifically that the energy of micro-system is converted into pressure, and then the pressure can be inputted to x, y, and z directions using system pressurization method. Roller pressure (i.e., compaction force) is exerted to the z direction simultaneously. MD simulation time is set to 30 ps because the micro-system has been stabilized through relaxation process. The adsorption process of carbon fiber to epoxy resin is shown in Figure 6.

### 3.4. Calculation of Enthalpy and Entropy

The entropy could be calculated based on the thermodynamic theory. When the entropy of micro-system within laying tows is converted from the initial state *S*_0_into *S_t_* under continuous laying loads, the difference value between their entropy values is shown in Equation (6).

(6) $$S_{t}-S_{0}=\int_{0}^{t}\frac{dQ}{T}$$

For a system that has *N* particles under temperature of *T*, the entropy could be expressed based on thermodynamic Boltzmann form.

(7) $$S_{t}=k\ln\Omega =k\ln q^{N}+NkT{\left(\frac{\partial \ln q}{\partial T}\right)}_{V^{\prime },N}$$

As described in Reference [35], the entropy *S*_0_ of particle swarm under the initial state of micro-system can be deduced as Equation (8).

(8) S0=3Nk{1−ln(ξωkT)}∫0ωLdωg(ω)

Furthermore, Equation (8) could utilize the normalization condition of quantum mechanics (Equation (9)).

(9) $$\int_{0}^{\omega_{L}}d\omega g(\omega )\;=\;1$$

In light of the above, low-entropy caused by high energy consumption is undesirable. Enthalpy represents all the energy per unit mass of the material. A certain quality of material could be driven from one state to another state under constant pressure during irreversible process, and then enthalpy increment would be tantamount to absorb the heat during this process. Therefore, enthalpy could be used to evaluate the consumption state of the internal energy that brings about molecular motion within the micro-system. The strain energy could only assess the magnitude and distribution of energy. Enthalpy and entropy are introduced to further assess the quality and characteristics of energy. According to Equation (5), enthalpy could be written as Equation (10):

(10) $$H=\kappa_{2}U_{c}+p{V^{\prime }}_{c}=NkT^{2}{\left(\frac{\partial \ln q}{\partial T}\right)}_{{V^{\prime }}_{c},N}+NkT{V^{\prime }}_{c}{\left(\frac{\partial \ln q}{\partial {V^{\prime }}_{c}}\right)}_{T,N}$$

**Figure 7:**
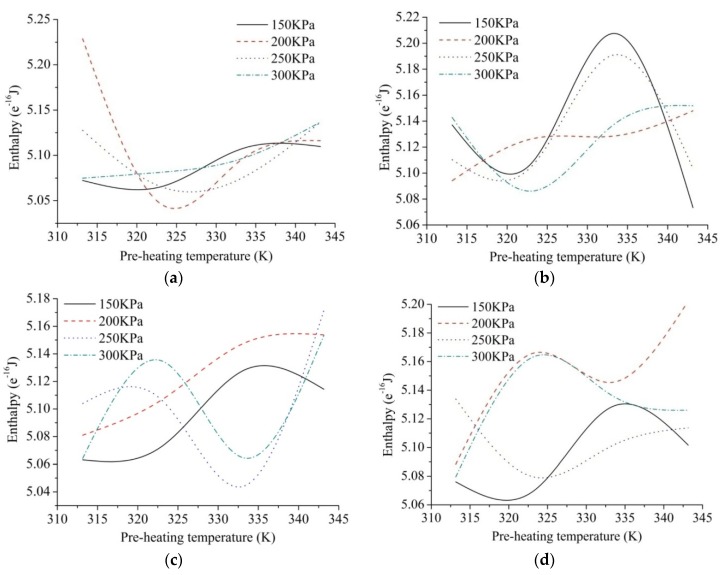
The fitting curves of enthalpy under different processing parameters: (**a**) 36 m/min; (**b**) 33 m/min; (**c**) 30 m/min; and (**d**) 27 m/min.

Based on Equations (8) and (10), entropy and enthalpy of micro-system could be calculated to assess energy properties and micro-mechanics properties under a certain equilibrium state. Quantitative description for energy quality and the internal energy of particle swarm could be carried out under different processing parameters during AFP process. With different laying speeds, enthalpy values exhibit different characteristics. The fitting curves of enthalpy are shown in Figure 7.

Figure 7a shows that the trends of enthalpy curves are from low to high with the rise of pre-heating temperature at a higher laying speed. In Figure 7b–d, most enthalpy curves have two inflection points with the rise of pre-heating temperature at the laying speed of 33 m/min, 30 m/min and 27 m/min, which could affect the variation trend of enthalpy. We also find that the internal energy of micro-system could not be improved with the increase of laying speed. Taken as a whole, the effect of pre-heating temperature and compaction force on the enthalpy is irregular, which indicates that there is a strong coupling interrelation among different processing parameters.

**Figure 8:**
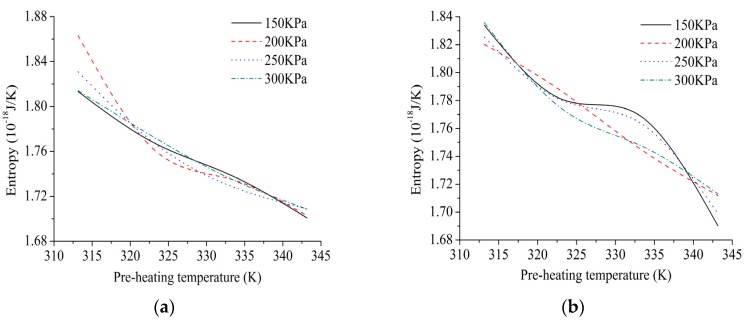

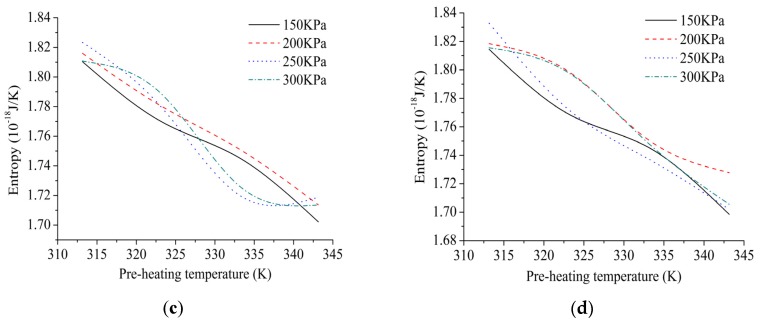
The fittingcurves of entropy under different processing parameters: (**a**) 36 m/min; (**b**) 33 m/min; (**c**) 30 m/min; and (**d**) 27 m/min.

The effects of different processing parameters on entropy should be identified with the changes of laying speeds. The fitting curves of entropy under different processing parameters are shown in Figure 8.

In Figure 8, effect of pre-heating temperature on the entropy of micro-system is more significant compared with laying speed and compaction force. The relationship between pre-heating temperature and the entropy could exhibit negative correlation. In other words, the higher the pre-heating temperature is, the lower the entropy is. Higher energy utilization efficiency and better energy quality could be realized at higher level of pre-heating temperature. The possible reason is that external heat source has done a reversible power to the system or exothermic process is driven during movement process. Therefore, in processing optimization, pre-heating temperature could be set to increase as much as possible within a reasonable range, which would guarantee low-entropy of micro-system for the laying tow. The result suggests that effect of compaction force and laying speed on the entropy could be ignored regarding the design of low-entropy, where only a single variable is considered.

## 4. Processing Optimization

In AFP process, some microscopic properties, such as adsorption of the fiber/matrix interface and fluidity of matrix along the fiber, are closely related to energy. The internal energy of micro-system, which results from the interaction of pre-heating process and external loads, is consistent with the laws of thermodynamics. Diffusion coefficients of matrix are calculated by utilizing the mean square displacement (MSD) and Einstein diffusion equation (Equation (11)) [36], which act as the analysis parameters of fluidity.

(11) $$\mathrm{MSD}=R(t)=\langle {|\vec{r}(t)-\vec{r}(0)|}^{2}\rangle =6\mathrm{Dt}$$

The fiber and the matrix are joined to form a whole composite by the interface of composites, which acts as a role of passing stress, enhancing compatibility of the fibers and the matrix. Thus, the mechanical properties of the composites can be determined by the strength of fiber/matrix interface directly. The adsorption energy (AE) acts as analysis parameter of interfacial adsorption, which is calculated by Equation (12) [37]:

(12) $$E_{\mathrm{interaction}}=E_{\mathrm{total}}-(E_{\mathrm{surface}}+E_{\mathrm{polymer}})$$

**Figure 9:**
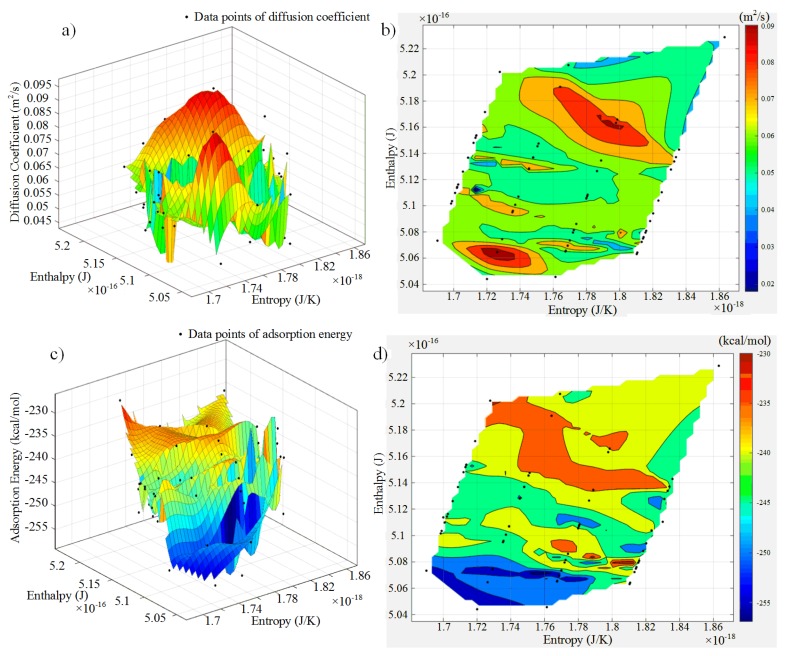
Relational surfaces and contour maps of micromechanics variables with enthalpy–entropy: (**a**) relational surface of diffusion coefficient with enthalpy–entropy; (**b**) contour maps of diffusion coefficient with enthalpy–entropy; (**c**) Relational surface of adsorption energy with enthalpy–entropy; and (**d**) contour maps of adsorption energy with enthalpy–entropy.

In this study, the diffusion coefficients of matrix and adsorption energy of interface are calculated using Equations (11) and (12) under different groups of processing parameters. Furthermore, two surfaces are cubically interpolated and fitted based on the calculation results of micromechanics, which combine with enthalpy and entropy. The fitting surfaces are shown in Figure 9a,c. To better understand their trends, their contour maps are plotted, which are shown in Figure 9b,d.

A better fluidity could be represented by a higher diffusion ability of epoxy resin. In addition, a good fluidity is beneficial to reducing pores precipitated by heat volatilization, due to movement and releasing of bubbles. Moreover, to relieve the tensile stress in the thickness direction and further reduce the longitudinal micro-cracks and delamination defects, strong interfacial adsorption is required in the selection of the processing parameters. These viewpoints are focused on in processing optimization of AFP. Thus, microscopic mechanical behavior and low-entropy design could be applied to engineering problems with multi-scale thought.

In Figure 9a,b, matrix has a good fluidity along the fiber in range of the enthalpy from 5.06 × 10^−16^ J to 5.08 × 10^−16^ J and the entropy from 1.7 × 10^−18^ J/K to 1.76 × 10^−18^ J/K, or the enthalpy from 5.16 × 10^−16^ J to 5.2 × 10^−16^ J and the entropy from 1.74 × 10^−18^ J/K to 1.82 × 10^−18^ J/K. In Figure 9c,d, characteristics of the surface are exhibited that the higher the enthalpy is, the higher the adsorption energy is. Moreover, interface has strong adsorption ability when the enthalpy range of the enthalpy is from 5.05 × 10^−16^ J to 5.09 × 10^−16^ J and the entropy falls in 1.70 × 10^−18^ J/K to 1.79 × 10^−18^ J/K. In summary, the enthalpy from 5.07 × 10^−16^ J to 5.08 × 10^−16^ J and the entropy from 1.72 × 10^−18^ J/K to 1.76 × 10^−18^ J/K could be chosen to guarantee better fluidity, stronger adsorption and higher energy quality simultaneously. In engineering field, this design method could avoid sophisticated optimization calculations, which could enhance the accuracy and reliability of processing optimization.

## 5. Experiments

### 5.1. Design and Process

In this section, defect states of laminates under the nine groups of processing parameters are assessed to further verify the effectiveness of the results obtained by simulation. The reasons are that some publications show that fluidity is closely related to voids [38,39]. Because the fluidity of resin along the fibers cannot be measured during AFP process, so we use void content as an important indicator to assess mechanical properties of laminates and the fluidity of resin. Bubble is one of the important causes of void defects. AFP process or curing process could result in the mechanical inclusion of air and the volatilization of volatile components subjected to the heat. The bubbles are further formed through the formation of internal nucleation. The bubbles usually appear at the fiber/matrix interface, which move with the flow of the resin. Under the same laying compaction force and curing pressure, the greater the flow rate is, the more rapid the movement of bubbles is, which have a great chance to spread to the air. On the other hand, voids and their distribution have a very negative effect on the interlaminar shear, compression and bending properties as well as the interlaminar stress level and the mechanical properties of the laminates [40]. In this paper, the validity of the processing parameters obtained by the simulation results is indirectly verified by comparing the void content of laminates made by different processing parameters.

The experimental scheme and purposes are designed and introduced as follows:

(1) The 6511 type carbon fiber/epoxy prepreg produced by Weihai Guangwei Composite Material Co., Ltd. in Weihai, China are selected as the experimental material. To avoid the effect of compaction force on the voids, the same compaction force is used in this experiment. Three levels of laying speed and pre-heating temperature are selected to compose the nine groups of experiments shown in Table 3.
(2) The experiments are carried out under different processing parameters using automated fiber placement machine.
(3) To avoid the effect of the curing process on the voids, the same curing processing parameters are used for curing, involving the curing pressure of 0.1 MPa, the curing temperature of 120 °C and curing time of 150 min. The voids are measured off-line using photographic method by optical microscope.

The experimental parameters are shown in Table 3.

**Figure 10:**
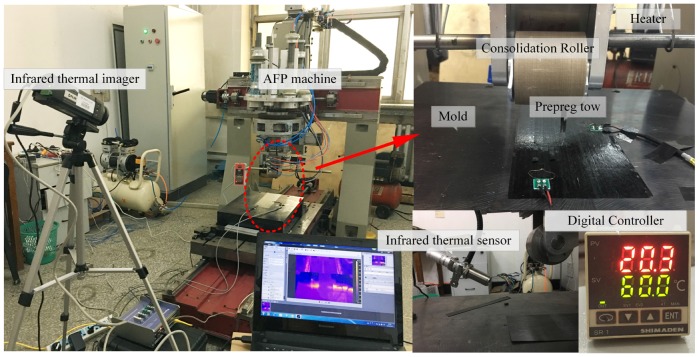
AFP machine and other equipment.

Gantry-type four-axis automated fiber placement machine is applied in the experiments, which is capable of automatic shearing, clamping and re-feeding for the prepreg tows. The precise control of pre-heating temperature required building a temperature control system that forms a temperature feedback and control loop through infrared probe (the range of 0–300 °C), relay, air switch and heater. Experimental AFP machine and other equipment are shown in Figure 10.

### 5.2. Results and Discussion

**Figure 11:**
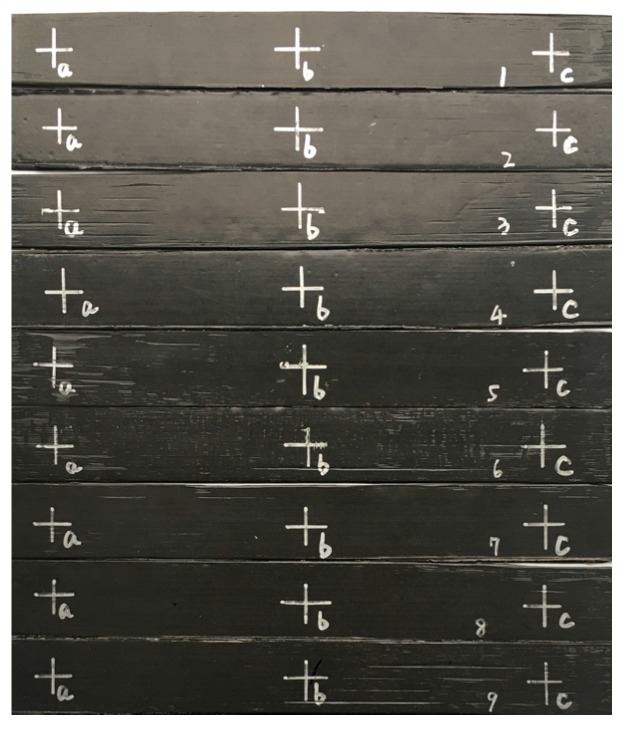
The nine groups of laminates.

The attenuation rate of three testing points, a, b and c, are measured by A-scan ultrasonic testing after curing. The off-line detection method, that is, photographic method by optical microscope, is used to quantify the void content of point c. The relationship between the void content and the attenuation rate is then gained. Thus, the other two testing points after curing could be estimated according to the attenuation rate. The average value of three points is used to evaluate the entire void content of the laminates before curing. One of the more accurate off-line defect detection methods is photographic method by optical microscope, which can detect the defect rate value less than 0.5%.In this paper, this method is used as the experimental calibration method. The c point is cut, and the detection profile is polished. Six fields of view are found under the microscope that observes in the profile of each sample. As the defects present dark, the gray value is marked by Image-pro, and the void content is further calculated. The average void content of the six fields of view is used to evaluate the true void content at point c. The nine groups of laminates after curing are shown in Figure 11.

**Figure 12:**
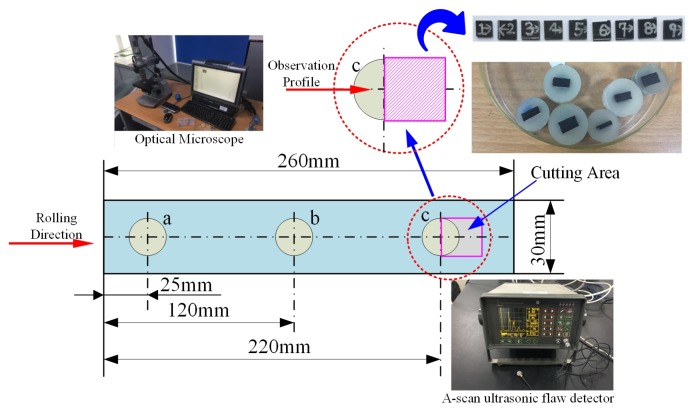
Off-line detection method.

The testing process is shown in Figure 12.

The average void contents of the different laminates are shown in the Table 4.

**Figure 13:**
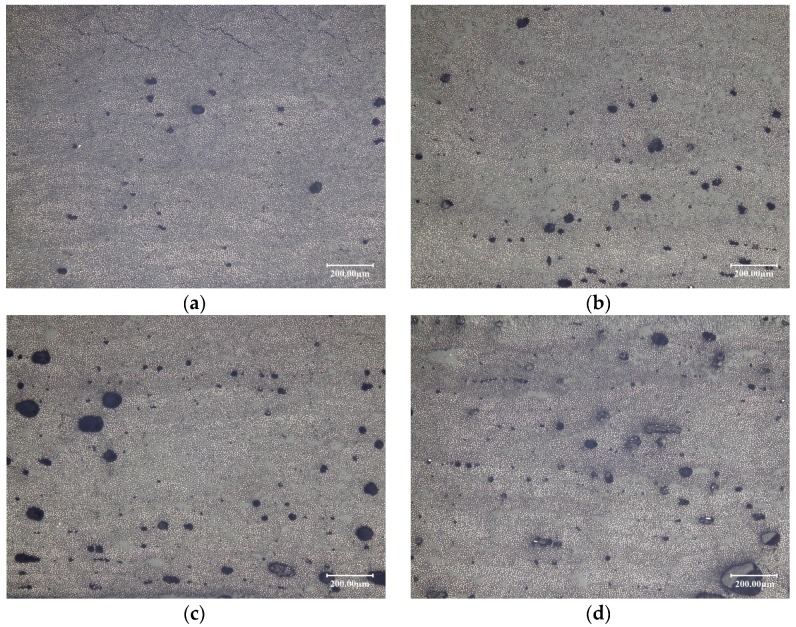
Manufacturing defects under the optical microscope: (**a**) 2-3; (**b**) 2-2; (**c**) 1-3; and (**d**) 3-1.

The defects under the optical microscope are shown in Figure 13.

As can be seen in Figure 7 and Figure 8, only the experimental group of 2-3 falls into the low-entropy region compared with other processing parameters. By comparing the void content of different laminates, the results show that the void content of 2-3 is the lowest, namely compaction force of 300 KPa, laying speed of 30 m/min and pre-heating temperature of 333.15 K, of which the void content can be less than 1%.The mechanical properties of the laminates are guaranteed, which verifies the effectiveness of the low-entropy optimization method. In addition, it can be seen from the experiment that the enthalpy is in the range from 5.07 × 10^−16^ J to 5.08 × 10^−16^ J, most of the void content of laminates can be guaranteed within 2%, such as 1-1 and 2-1. Therefore, if some limitations are caused by the equipment or processing, which cannot choose the low-entropy design region of the processing parameters, the enthalpy from 5.07 × 10^−16^ J to 5.08 × 10^−16^ J could be chosen to ensure a lower level of defect rate.

## 6. Conclusions

A novel multi-scale low-entropy method for optimizing processing parameters is proposed to guarantee the cooperativity of multi-scale effect, energy consumption, energy utilization efficiency and mechanical properties. Taking carbon fiber/Bisphenol A epoxy prepreg as an example, mechanical properties of different scales under different processing parameters are calculated, wherein multi-scale energy transfer model is established and used to communicate with different scales. Low-entropy region is identified to assess energy properties and quality with micro-system. Some experiments are then conducted to verify the feasibility of the processing optimization method. Some conclusions are drawn as follows.

(1) The peak of enthalpy curves raise from low to high with the rise of pre-heating temperature at higher laying speed. In addition, most enthalpy curves have two inflection points with the rise of pre-heating temperature at the laying speed of 33 m/min, 30 m/min and 27 m/min. The effect of pre-heating temperature and compaction force on the enthalpy is irregular because of strong coupling interrelation among different processing parameters.
(2) According to the fitting curves of trend of entropy under different processing parameters, effect of pre-heating temperature on the entropy of micro-system is more significant compared with laying speed and compaction force. Due to reversible power by external heat source or exothermic process during movement process, relationship between pre-heating temperature and the entropy could exhibit negative correlation. The other processing parameters have little effect on the entropy of micro-system.
(3) Low-entropy region is found, namely the enthalpy from 5.07 × 10^−16^ J to 5.08 × 10^−16^ J and the entropy from 1.72 × 10^−18^ J/K to 1.76 × 10^−18^ J/K, which are chosen to guarantee better fluidity, stronger adsorption and higher energy quality simultaneously.
(4) Experimental results show that the void content of the laminate made by processing parameters within the low-entropy region is lower. In addition, if the enthalpy is in the range from 5.07 ×10^−16^ J to 5.08 ×10^−16^ J, most of the void content of laminates can be guaranteed within 2%.

Different curing techniques should also be considered to improve the theoretical model in future work.

## Figures and Tables

**Table 1:** Mechanical properties of a Bisphenol A epoxy matrix prepreg.

Density	E1	E2, E3	v12, v13	v23	G12, G13	G23
1.49 g/cm^3^	121 GPa	76 MPa	0.27	0.4	4.7 GPa	49 MPa

**Table 2:** Boundary conditions of micro-system.

**BoundaryParameters**	The Number of Particles	ω	The Area of Meso-Unit
**Value**	3096	4.62 × 10^12^	2.12
**unit**	-	Hz	mm^3^
**Boundary parameters**	The area of micro-system	S_0_	*κ*_2_
**Value**	35.8	4.03 × 10^−^^19^	1.69 × 10^−^^17^
**unit**	nm^3^	J/K	-

where *ω* is calculated by using quantum harmonic oscillator theory.

**Table 3:** Experimental parameters (Compaction force is 300KPa).

Level	1	2	3
**Laying speed**	27 m/min	30 m/min	33 m/min
**Pre-heating temperature**	313.15 K	323.15 K	333.15 K

**Table 4:** The average void contents of the different laminates.

Experimental Group	1-1	1-2	1-3	2-1	2-2	2-3	3-1	3-2	3-3
**Void content (%)**	1.148	2.772	3.057	1.275	1.561	0.947	3.193	4.678	2.118

Note: “1-1” represents experimental group under the first level of laying speed and the first level of the preheating temperature, see Table 3.

## Nomenclature

E1, E2, E3	Modulus of elasticity in the x, y, z directions respectively (x is the length direction of tow, y is the width direction of tow, z is the vertical direction), MPa or GPa.
v12, v13, v23	Poisson’s ratio in different planes (xy, xz, and yz planes, respectively).
G12, G13, G13	Shear modulus in different planes (xy, xz, and yz planes, respectively), MPa or GPa
*ε_i_*	Strain component, *i* = 1, 2, 6
*C_ijkl_*	Stiffness coefficient, *i*, *j*, *k*, *l* = 1, 2, 6
*σ_i_*	Stress component, *i* = 1, 2, 6, Pa
*C_ij_*,*C_ijk_*,*C_ijkl_*	Stiffness tensors*, i*, *j*, *k*, *l* = 1, 2, 6
*dU*	Internal energy increment, J
*u_o_*	Strain energy density, J/m^2^
*V*	Volume of meso-unit, μm^3^
*Q*(*U*)	Total strain energy, J
*U_m_*	The internal energy of a certain meso-unit, J
*V_m_*	The volume of a certain meso-unit, μm^3^
*κ*_1_	The energy scaling of macro–meso
*κ*_2_	The energy scaling of meso–micro
*U_c_*	The accumulated internal energy in No.C meso-unit, J
*Uʹ_c_*	The energy of a certain micro-system within No.C meso-unit, J
*V_c_*	The volume of No.C meso-unit, μm^3^
*Vʹ_c_*	The volume of a certain micro-system within No.C meso-unit, nm^3^
*S*_0_	The entropy value of the initial state within micro-system, J/K
*S_t_*	The entropy value of the equilibrium state at time of *t*, J/K
*T*	The temperature of the external heat source, K
*dQ*	The heat energy absorbed by the system when it comes into contact with a heat source with a temperature of *T*, J
Ω	The number of micro-states
*q*	The sum of effective quantum states
*ξ*	*h*/2*π*
*h*	Planck’s constant
*k*	Boltzmann’s constant
*w*	The vibration frequency of particle swarm, Hz
*ω_L_*	The vibration frequency of particle in the space of *L*, Hz
*g*(*ω*)	The distributionfunction of *ω*
*p*	The pressureofthe micro-system, MPa
*N*	The number of particles in the micro-system
D	Diffusion coefficient
*r*(t)	The atomic position in the moment of t
*r*(0)	The original position of atom, m^2^/s
*E*_interaction_	Adsorption energy, J
*E*_total_	Total energy of micro-system, J
*E*_surface_	Energy of fiber layer, J
*E*_polymer_	Energy of matrix layer, J
